# Automated MAD and MIR structure solution

**DOI:** 10.1107/S0907444999000839

**Published:** 1999-04-01

**Authors:** Thomas C. Terwilliger, Joel Berendzen

**Affiliations:** a Structural Biology Group, Mail Stop M888, Los Alamos National Laboratory, Los Alamos, NM 87545, USA; b Biophysics Group, Mail Stop D454, Los Alamos National Laboratory, Los Alamos, NM 87545, USA

**Keywords:** MAD, MIR, automated structure solution

## Abstract

Obtaining an electron-density map from X-ray diffraction data can be difficult and time-consuming even after the data have been collected, largely because MIR and MAD structure determinations currently require many subjective evaluations of the qualities of trial heavy-atom partial structures before a correct heavy-atom solution is obtained. A set of criteria for evaluating the quality of heavy-atom partial solutions in macromolecular crystallography have been developed. These have allowed the conversion of the crystal structure-solution process into an optimization problem and have allowed its automation. The *SOLVE* software has been used to solve MAD data sets with as many as 52 selenium sites in the asymmetric unit. The automated structure-solution process developed is a major step towards the fully automated structure-determination, model-building and refinement procedure which is needed for genomic scale structure determinations.

## Abstract 2

A fully automated procedure for solving MIR and MAD structures has been developed using a scoring scheme to convert the structure-solution process into an optimization problem.

## 1. Introduction

Recently, the pace of macromolecular structure determination by X-ray crystallography and NMR has seen a rapid acceleration. In 1990 just 164 new macromolecular structures were added to the Protein Data Bank (Bernstein *et al.*, 1977 ▶), but by 1997 this had increased tenfold to 1640. At the same time that the rate of obtaining new structures has been increasing, the time required to obtain a particular new structure has decreased. Within the past year, there have been a number of cases in which a protein crystal structure has been solved within one day of collecting X-ray data (*e.g.* V. Ramakrishnan, personal communication; R. Fahrner & D. Eisenberg, personal communication; S.-H. Kim, personal communication, R. Stevens, personal communication). While the current pace of macromolecular structure determination is impressive, it will require much greater throughput if it is to ever be applied on a scale which compares with the genomic sequencing projects now under way. If macromolecular structures could be determined at even more rapid rates, it would become possible to determine structures of broad groups of proteins on a genomic scale (*e.g.* Pennisi, 1998 ▶; Rost, 1998 ▶; Shapiro & Lima, 1998 ▶; Terwilliger *et al.*, 1998 ▶).

### 1.1. ‘Solving’ structures using MAD or MIR X-ray data

One of the limiting stages in macromolecular structure determination by X-ray crystallography can be ‘solving’ the structure using multiple isomorphous replacement (MIR) or multiwavelength anomalous scattering (MAD) X-ray data (Ke, 1997 ▶; Hendrickson & Ogata, 1997 ▶). This stage of structure solution is often difficult because the partial structures of the heavy or anomalously scattering atoms which have to be solved in the MIR and MAD methods can be very complicated. Furthermore, it can be both time-consuming and challenging to identify and verify these partial structures. Structure solution by MIR or MAD currently involves many steps which require decisions to be made by the crystallographer and requires operation of several computer programs or different parts of a software package to carry out. If this process could be carried out in an automated fashion, the time required to solve a macromolecular structure once data has been collected might be greatly reduced. Despite the complexity of the MIR or MAD structure-determination process, each of the individual steps is well defined, and most possible outcomes and decisions which must be made can be anticipated in advance. Additionally, suitable computational algorithms exist for every stage in the process. This means that a complete automation of the structure-determination process is achievable, at least in principle.

The MIR and MAD structure-determination procedures are closely related and have several critical steps in common. Two of these are the identification of possible partial structures of the heavy or anomalously scattering atoms in the structure and the evaluation of the quality of each of these solutions. In both the MAD and MIR methods, possible partial structures of the heavy or anomalously scattering atoms are generally obtained either by manual or semi-automated inspection of difference Patterson functions (Terwilliger *et al.*, 1987 ▶; Chang & Lewis, 1994 ▶; Vagin & Teplyakov, 1998 ▶) or by direct methods (Sheldrick, 1990 ▶; Miller *et al.*, 1994 ▶). For example, a semi-automated procedure (‘*HASSP*’) which is widely used for generating possible partial structures is based on the superposition method of Buerger (1970 ▶) and yields a ranked list of partial structures which are compatible with the difference Patterson function (Terwilliger *et al.*, 1987 ▶). Such a list of potential solutions to the difference Patterson is only a starting point in either the MAD or MIR methods, however, as each potential partial structure must then be individually completed and evaluated.

In the MAD method, the trial anomalously scattering atom partial structure is generally refined and used to identify further anomalously scattering atoms by difference Fourier (or anomalous difference Fourier) analysis. The completed partial structure is then used to calculate phases for the entire structure, and the resulting electron density is examined visually to determine if it has the features expected of the macromolecule. This visual examination is crucial for determining whether the entire process has been successful, but there are several criteria which are commonly used at earlier stages to determine whether the structure-determination process is going well. These include the compatibility of the partial structure with the anomalous or dispersive difference Patterson functions, the figure of merit of phasing and the appearance of anomalously scattering atom sites in difference Fourier analyses calculated after omitting these sites in phasing.

The process of completing a trial heavy-atom partial structure in the MIR method differs slightly from that used in the MAD approach because the partial structures of heavy atoms must generally be determined in more than one heavy-atom derivative. Starting solutions for heavy-atom partial structures can usually be obtained for each of the available heavy-atom derivatives. These trial partial structures are ordinarily then refined and used to calculate phases for the native structure. The native phases are in turn used to calculate difference Fouriers for the other derivatives in order to identify possible heavy-atom sites in those derivatives. Additional heavy-atom sites identified in this way are included in the phasing, and the process is repeated until no further sites are found. As in the case of MAD structure determination, the structure is generally considered solved when the resulting native electron-density map is interpretable by the crystallographer. Indications that the structure determination is proceeding well are similar to those used in MAD structure determination. They include the compatibility of each heavy-atom partial structure with the corresponding difference Patterson function, the figure of merit of the phasing and cross-difference Fourier analyses involving the use of one set of derivatives in the phasing calculation and calculating a cross-difference Fourier for a different derivative.

### 1.2. Automated decision-making during structure determination

There are several important decisions which must be made by the crystallographer during structure solution by either the MAD or MIR methods. At early stages in the process, a key decision is to choose which trial partial structures are worth pursuing further. At later stages, key decisions must be made as to whether a particular peak found in a difference Fourier analysis should be included as part of the heavy-atom partial structure or not and which hand of the heavy atoms is correct. In the final stages of structure determination, key decisions include the decision as to which of the possible partial structures is most likely to be correct and whether the structure-solution process is completed.

An important aspect of the present work is the recognition that all these decisions could be made in a uniform way if a suitable scoring algorithm could be developed. With a scoring procedure, the decision-making process with incompletely defined criteria described above becomes instead an optimization process with a well defined target function. For example, if a list of trial heavy-atom or anomalously scattering-atom partial structures could be scored in a useful way and ranked, then the highest-scoring partial structures at each stage of the analysis would be most likely to be correct and could be pursued more aggressively than lower-scoring solutions. Additional sites would be included in a partial structure and the inverse heavy-atom partial structure would be used if doing so increased the score. The structure-determination process would be completed when no partial structures with higher scores than those of the current set could be obtained. Based on this analysis, we propose that the development of a comprehensive scoring procedure for heavy-atom partial structures could make the process of structure determination well defined and amenable to automation. In this, we describe such a scoring system and the resulting fully automated system (‘*SOLVE*’) for macromolecular structure determination by the MIR or MAD approaches.

## 2. Materials and methods

### 2.1. Evaluation of the match between a heavy-atom partial structure and a Patterson or difference Patterson function

The first criterion we use for evaluating a trial heavy-atom solution is whether the Patterson function calculated from a heavy-atom partial structure matches the observed Patterson or difference Patterson function. This has always been an important criterion in the MIR and MAD methods (Blundell & Johnson, 1976 ▶). Our scoring in this case essentially consists of the average value of the Patterson function at predicted locations of peaks, multiplied by a weighting factor based on the number of heavy-atom sites in the trial solution. The complete raw score 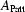 for a match between the Patterson function and a trial solution is given by 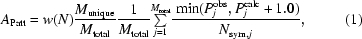
where there are 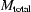 predicted interatomic vectors in the Patterson function for the trial partial structure. In this calculation, the Patterson or difference Patterson function is first normalized to its r.m.s. value. Then peaks which occur in regions of the Patterson function where 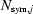 symmetry-related interatomic vectors coincide are divided by this symmetry number 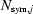 (Terwilliger *et al.*, 1987 ▶). To exclude contributions from very high peaks which are unlikely to correspond to interatomic vectors in the model, occupancies of each heavy-atom site are refined so that the predicted peak heights 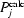 match the observed peak heights 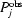 at the predicted interatomic positons as closely as possible. All peak heights more than 1σ higher than their predicted values are then truncated at this height. The average value of the Patterson function at predicted interatomic vectors estimated in this way is then corrected for instances where several predicted vectors unrelated by symmetry fall on the same location by scaling it by the fraction of predicted vectors which are unique, 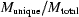. Finally, a weighting function *w*(*N*) (see below) is applied to this average value to give the raw Patterson score.

### 2.2. Calculation of cross-validation difference Fourier maps

The second criteria used to evaluate heavy-atom solutions is whether each heavy-atom site appears in a ‘cross-validation’ difference Fourier analysis calculated after omitting this site (and all equivalent sites in other derivatives) from the phase calculation. A related approach in which one derivative is omitted from phasing and the other derivatives are used to phase a difference Fourier has been used for some time (Dickerson *et al.*, 1961 ▶). Our raw score for cross-validation difference Fouriers is the average peak height calculated in this way for each heavy-atom site, multiplied by the weighting function *w*(*N*) described below.

### 2.3. Weighting function for Patterson and cross-validation difference Fourier scores

Our unweighted raw scores for evaluation of Patterson and cross-validation difference Fouriers are based simply on average peak height. It seems likely that in most cases, if two solutions are being considered and they have equal average peak heights but differing numbers of heavy-atom sites, the solution with the larger number of sites is more likely to be correct. On the other hand, just how to weight this increase in number of sites is not clear. If the average peak height is simply multiplied by the number of sites, then solutions with very low average peak heights can receive high scores, for example. We have chosen an intermediate ground. The weighting function *w*(*N*) we use for the cross-validation difference Fourier is designed to favor the addition of a new site to an existing partial structure with *N* − 1 sites as long as the average value of the peaks at the additional sites is at least a fraction 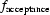 of the average for the existing sites. A weighting function which has this property is given by 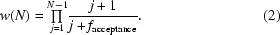
This weighting function is applied to both the Patterson and cross-validation difference Fourier scores. In the case of the Patterson function, there are generally more predicted interatomic vectors 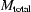 than heavy-atom sites *N*, but we use *N* in the calculation of the weighting factor *w*(*N*) so as to make the weighting the same for Patterson and Fourier scoring. The parameter 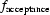 is ordinarily set at a level of 0.2–0.35, so that additional sites which yield cross-validation difference Fourier peak heights 1/5 to 1/3 of the average would just be included in the heavy-atom model.

### 2.4. Evaluation of figure of merit of phasing

An important criteria for evaluating the quality of phasing in both the MAD and MIR methods is the overall figure of merit *m* (Blundell & Johnson, 1976 ▶). This parameter is sensitive to errors in heavy-atom occupancies, to the resolution of the data and to the method used to calculate phases. Nevertheless, if a single procedure is used consistently then it can be used to distinguish between solutions which have more or less potential for accurate phasing. Additionally, in the *SOLVE* procedure, heavy-atom occupancies are refined by origin-removed Patterson refinement, which has been demonstrated to yield relatively unbiased estimates of occupancy (Terwilliger & Eisenberg, 1983 ▶). The raw score by this criteria is simply the unweighted average figure of merit for all reflections included in phasing.

### 2.5. Evaluation of distinction between solvent and macromolecule in native Fourier

The final criteria used in our scoring procedure is whether the native Fourier (electron-density map) calculated based on the trial heavy-atom solution has the features expected of a crystal of a macromolecule. We have focused on one such feature which is relatively simple to evaluate, namely whether the map has distinct regions of solvent and macromolecule (Terwilliger & Berendzen, 1999 ▶). Our measure of this distinction is the variation, from one location to another in the native Fourier, of the r.m.s. electron density (not including the 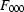 term in the Fourier synthesis). In regions which contain solvent, the native Fourier is flat and the r.m.s. electron density calculated in this way is very low. In regions containing the macromolecule, the native Fourier has many peaks and valleys and the r.m.s. electron density is high. A map with a clear definition of solvent and macromolecule will have a high variation of local r.m.s. electron density from location to location in the map. The raw score for this criteria is the standard deviation of the local r.m.s. electron density calculated in boxes with dimensions approximately twice the resolution of the map in each direction.

We have shown elsewhere (Terwilliger & Berendzen, 1999 ▶) that a score of this type calculated from the native Fourier can be an excellent indicator of the quality of the map when the map is of moderate or better quality. Based on model calculations, this score is useful when the mean phase error for the map is about 80° or less. This corresponds roughly to a figure of merit of phasing of about 0.2 or greater.

### 2.6. Calculation of final score for a heavy-atom partial structure

The overall scoring procedure is in three steps. A starting set of 10–50 trial heavy-atom partial structures are each given raw scores based on each of the four criteria described above and shown in Table 1 ▶. The mean and standard deviation of the raw scores for each criterion are calculated and are then used as a basis for normalizing all these and later raw scores to yield *Z* scores for each criteria, where the *Z* score, based on a raw score of *A* and a mean and standard deviation for the starting set of 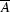 and 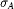, is given by 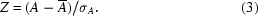
The final score for a heavy-atom solution is the sum of the *Z* scores for each of the four criteria. To reduce the likelihood of obtaining a high-scoring solution based on just the Patterson, figure of merit or cross-validation difference Fourier *Z* scores, the final score is adjusted by subtraction of half the differences between each of these and lowest *Z* score among them.

When the native Fourier is of low quality, the corresponding score is not of significant utility. To reduce the contribution of the scoring from the native Fourier in cases where it is not expected to be of value, we limit the *Z* score for the native Fourier to a maximum value depending on the figure of merit of the map. The maximum value is set at the value obtained for cases with the corresponding figure of merit in a series of model calculations we carried out using selenomethionine MAD data and the gene 5 protein atomic model (Terwilliger & Berendzen, 1999 ▶; Skinner *et al.*, 1994 ▶). These model cases resulted in the approximate relation 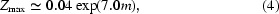
where *m* is the average figure of merit of the phase calculation. That is, for a map with a figure of merit of 0.4, the maximum *Z* score allowed for this criteria would be just 0.6, while for a map with a figure of merit of 0.6 it could be as high as 2.7.

### 2.7. Automated MIR and MAD structure determination

**Figure 1:**
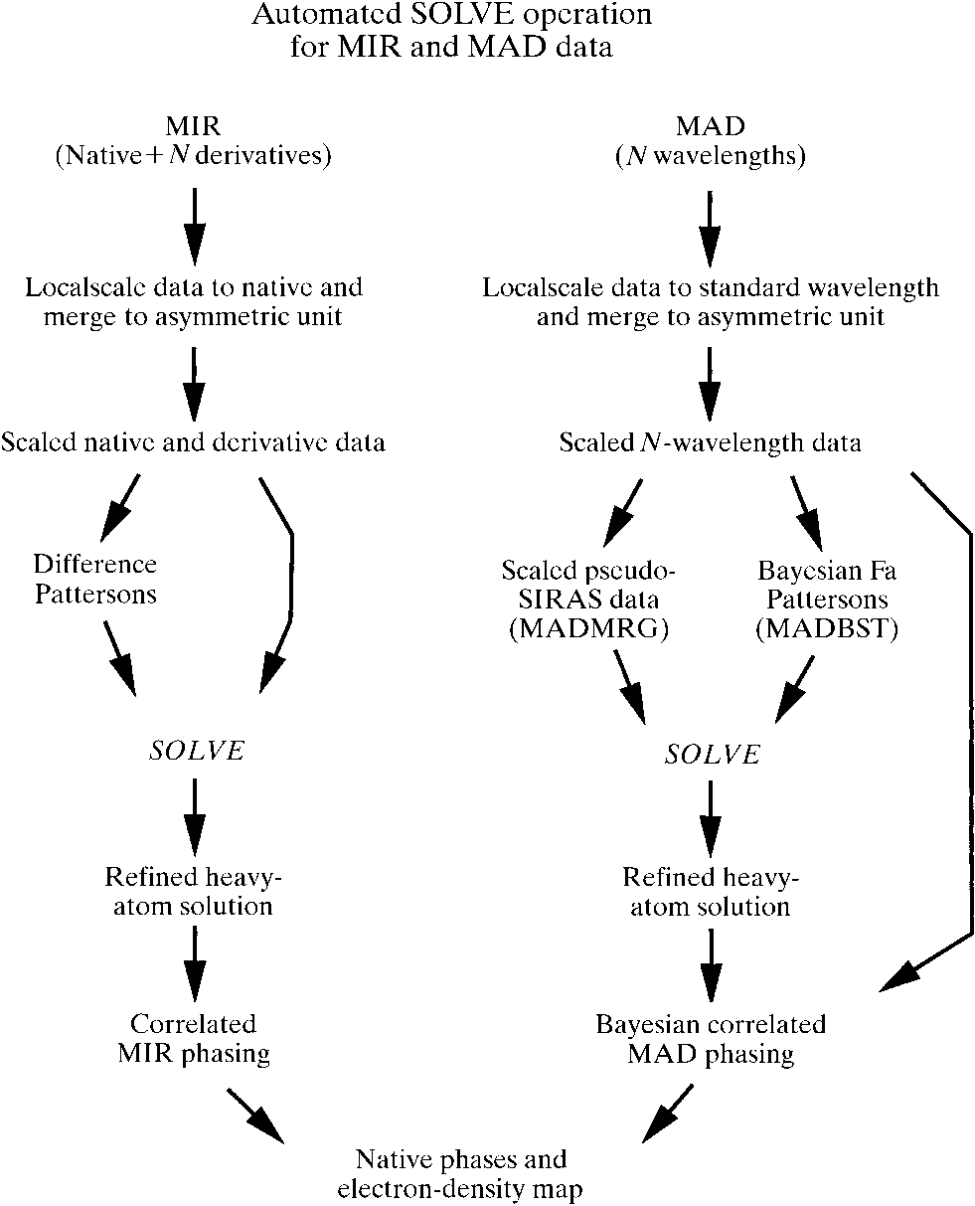
Steps in automated structure determination by *SOLVE*.

Fig. 1 ▶ outlines the main steps carried out by the automated ‘*SOLVE*’ procedure for MIR and MAD structure determination. These consist of scaling the data, calculation of Patterson functions, finding and optimizing the heavy-atom partial structure and calculating native phases and an electron-density map. The procedures for MAD and MIR data are very similar, except that the MAD data is scaled slightly differently from MIR data and the MAD data is converted to a pseudo-SIRAS (single isomorphous replacement with anomalous scattering) form before looking for the anomalously scattering-atom partial structure. This conversion allows heavy-atom refinement, which would otherwise be prohibitively slow, to be carried out very quickly by Patterson-based refinement (Terwilliger & Eisenberg, 1983 ▶; Terwilliger, 1994*b* ▶). Each of these steps is described in detail below.

### 2.8. Scaling of X-ray data sets

The *SOLVE* procedure begins with integrated scaled or unscaled X-ray intensities from several X-ray wavelengths (for MAD data) or for native and several heavy-atom derivative structures (for MIR data), such as those produced by *HKL* (Otwinowski & Minor, 1997 ▶), *MOSFLM* (Leslie, 1993 ▶) or d*TREK (J. Pflugrath, personal communication). In either the MAD or MIR cases the raw intensities are converted to structure-factor amplitudes, which are brought to a common scale and partially corrected for absorption and decay effects using a local scaling procedure (Matthews & Czerwinski, 1975 ▶). The overall strategy for scaling is to minimize systematic errors by scaling 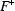 and 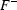 in as similar a fashion as possible and by keeping different data sets separate until after scaling is completed. The scaling procedure used by *SOLVE* is optimized for cases where data are collected in a systematic fashion so that, for example, the reflections measured for each wavelength of a MAD experiment are nearly identical.

### 2.9. Scaling of MIR data sets

The scaling of MIR data sets is straightforward in *SOLVE*. The general approach is to scale the native data, then to use it as a reference dataset for scaling of the 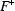 and 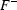 data from each derivative and finally to merge all the data together.

### 2.10. Scaling native data

Ordinarily, the raw native data suitable for *SOLVE* analysis consists of one or more individual files each containing measurements of reflection intensities obtained by rotation of a crystal by 180° or less about an axis. In this way, all but a few high-resolution *F*(*h*,*k*,*l*) are present at most once in an individual file, and the data can be handled as if each point on the reciprocal lattice either has an observation associated with it or not. If the data are collected by rotations of more than 180°, the data can be broken up into smaller files for analysis.

The native data is scaled in three steps. In the first step, a reference data set is constructed from a file containing native data from a single experiment. The reference data set is constructed by local-scaling this data set to itself as follows. For each reflection (*h*,*k*,*l*) in the asymmetric unit, all amplitudes of structure factors equivalent by space-group symmetry are averaged to yield a merged reduced data set. This data set is then expanded to the entire reciprocal lattice using space-group symmetry and assuming |*F*(*h*,*k*,*l*)| = |*F*(−*h*,−*k*,−*l*)|. This yields an averaged data set which has exact symmetry. The raw data are then local-scaled to this averaged data set. Local scaling is carried out in *SOLVE* one reflection (*h*,*k*,*l*) at a time. The average structure-factor amplitude for at least 30 reflections symmetrically arranged around (*h*,*k*,*l*) in reciprocal space is obtained using the same (*h*,*k*,*l*) for the raw and averaged data sets. The scale factor applied to *F*(*h*,*k*,*l*) for the raw data is then the ratio of these averages. The local-scaled raw data are then reduced to the asymmetric unit and duplicates are averaged to yield a scaled native data set.

The second step in scaling the native data is to place the reference data set on an approximate absolute scale. Setting the absolute scale of the data is helpful for several of the procedures used by *SOLVE*. For example, if the scale of the data is known, then occupancies of heavy-atom sites can be reasonably be expected to be in the range of about 0.1–1.0. The reference data set is placed on a very approximate absolute scale using information on the number of amino-acid residues in the macromolecule (if it is a protein) along with the mean intensity of reflections in the lowest resolution shell. This simple approach is used rather than a Wilson plot (Wilson, 1942 ▶) so that the same algorithm can be applied for either low-resolution or high-resolution data.

The final step in scaling the native data is to scale all the available native data to the reference data set and then to reduce all the scaled data to the asymmetric unit and merge it into a single native data set.

### 2.11. Scaling of derivative data

Derivative data is scaled to the native data set after first separating the 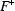 data from the 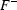 data. The 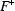 and 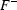 data are each scaled to the native data set using local scaling. The 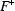 and 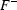 data are then reduced to the asymmetric unit, averaging measurements of equivalent reflections. Finally, two scaled data files are constructed. Each contains the scaled native data 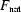 and 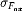. One also contains 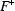, 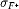, 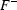 and 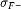 for each derivative and the other contains the average amplitude 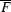, 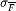 and the anomalous difference 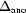, 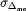 for each derivative.

### 2.12. Scaling of MAD data

MAD data is analyzed a little differently from MIR data by *SOLVE* because there is no native data set to use as a reference for all the data. The general approach used is to combine all available data into one reference data set, then to separate out Bijvoet pairs and to scale each individual 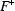 or 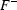 data set to the reference data set. The scaling is performed in two stages, with each individual 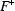 or 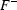 data set first scaled to the first data set with an overall scale factor and *B* factor so as to put all the data sets on the same scale. Then all data in all data sets are merged to the asymmetric unit and averaged to form the reference data set. Finally, each individual 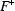 or 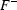 data set is scaled to the reference data set with local scaling. This scaling method is used by version 1.10 of *SOLVE*. Earlier versions (including ones used in this paper) used a more complicated approach, in which each 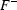 set of data was first scaled to 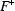 at each wavelength and then all the wavelengths of data were scaled together. The approach described here is now used because it is simpler and yields *R* factors that are equal to or lower than those obtained with the more complicated approach.

### 2.13. Calculation of Patterson and difference Patterson functions

*SOLVE* uses Patterson or difference Patterson functions to generate and evaluate plausible heavy-atom solutions in MIR and MAD data sets. In the case of MIR (or SIRAS, single isomorphous replacement with anomalous scattering) data, the differences between each derivative and the native are used to calculate difference Patterson functions which serve as a starting point for obtaining possible heavy-atom partial structures. In the case of MAD data, the multiwavelength data are combined to yield Bayesian estimates of the amplitude 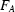 and relative phase α of the structure factor corresponding to the anomalously scattering atoms. These structure-factor amplitudes are in turn used to calculate a Patterson function corresponding to the partial structure of the anomalously scattering atoms (*MADBST*; Terwilliger, 1994*a* ▶). Additionally, the multiwavelength data are used to generate a pseudo-SIRAS data set which is then treated just like an SIRAS data set until the final stage of phase calculation (*MADMRG*; Terwilliger, 1994*b* ▶).

### 2.14. Solving the heavy-atom structure

The core of the *SOLVE* algorithm is the identification and optimization of the heavy-atom (or anomalously scattering-atom) locations, occupancies and thermal parameters. MAD and MIR data sets are treated identically for this part of structure determination. This is possible because MAD data has been converted to pseudo-SIR data with anomalous differences in the previous step. In either the MAD or MIR cases, the available data consist of a Patterson function for each ‘derivative’ (where there is a single ‘derivative’ for MAD data) and scaled data for a ‘native’ and one or more ‘derivatives’.

**Figure 2:**
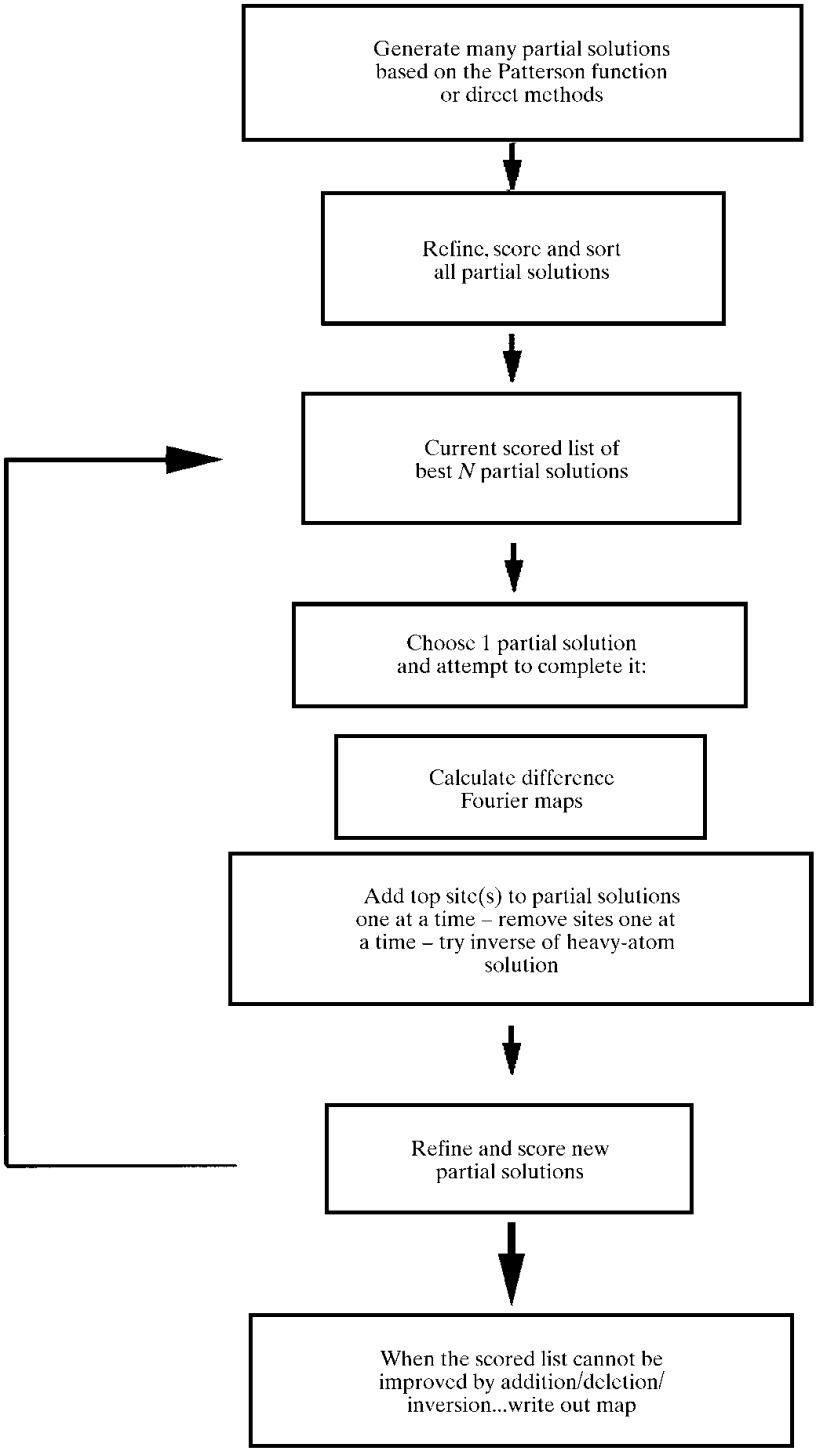
Determining the heavy-atom partial structure in *SOLVE*.

Fig. 2 ▶ illustrates the approach used by *SOLVE* for determining the heavy-atom structure. The procedure begins by generating a few likely partial solutions to the heavy-atom structure which are then used as ‘seeds’ to generate more complete solutions. The generation of seeds is carried out by construction of a list of trial partial solutions for the heavy-atom structure using *HASSP* (Terwilliger *et al.*, 1987 ▶), followed by refinement and scoring of each trial solution. The top seeds (typically five) are then used in the generation of new trial solutions by addition and refinement of sites identified by difference Fourier analysis, subtraction of sites and by inversion. The last step is carried out iteratively until no further improvement is obtained. The scoring procedure described above is used to identify those trial solutions which are likely to be correct, and at each stage a group of solutions with high scores is maintained.

### 2.15. Obtaining potential seeds using *HASSP*

Trial partial solutions (‘seeds’) for the heavy-atom structure can be input directly to *SOLVE*, but are generally obtained by analysis of the Patterson function using the automated procedure *HASSP* (Terwilliger *et al.*, 1987 ▶). This procedure uses the superposition method (Buerger, 1970 ▶) for deconvolution of a Patterson function, and it scores solutions based on the likelihood of obtaining the solution by chance. *SOLVE* then calculates a preliminary score for each of these solutions based on the Patterson function alone as described above. *SOLVE* analyzes the Patterson or difference Patterson functions for each of the derivatives which are being considered, and chooses the top solutions from each derivative as potential seeds.

### 2.16. Refinement and scoring of potential seeds

Potential seeds are refined using origin-removed Patterson refinement as implemented in the program *HEAVY* (Terwilliger & Eisenberg, 1983 ▶). This procedure for heavy-atom refinement has three features which are critical to *SOLVE*. One is that the occupancies, thermal factors and positions can be refined with origin-removed Patterson refinement using a single derivative. This means that the MAD data which is converted to a pseudo-SIRAS form can be refined effectively. The second feature is that this procedure yields relatively unbiased estimates of occupancies. This is important as it means that occupancies are not systematically overestimated when the data is poor, so that the overall figure of merit is a relatively good indication of the phasing quality. The third important feature is that Patterson-based refinement is fast, as derivatives are independent of each other and phases only need to be calculated every few cycles. This speed is crucial to the operation of *SOLVE* because even so as much as 75% of the time running *SOLVE* is spent on heavy-atom refinement and phasing.

Potential seeds are rejected in the heavy-atom refinement step if the refinement does not yield plausible parameters. For example, any seed for which occupancies of all sites refine to zero, for which coordinates shift by large distances, for which the figure of merit is low (less than 0.01) or for which heavy-atom refinement fails for any reason is rejected.

Once the heavy-atom parameters in a potential seed have been refined, the solution is scored using the four criteria in Table 1 ▶. The top group of solutions is then used as seeds in the next step, described below.

### 2.17. Generating new trial solutions

*SOLVE* generates new trial solutions in three ways: by addition of sites identified from difference Fourier analysis, by deletion of sites and by inversion. For example, a seed obtained as above is used to calculate native phases, and from these phases difference Fourier maps are calculated for each derivative. In the case of MAD data, the difference Fourier maps are calculated using the native phases along with the 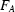 and α values for the anomalously scattering partial structure estimated from *MADBST* (Terwilliger, 1994*a* ▶). The top peaks in the difference Fourier maps are added to the seed one at a time in order to generate new trial solutions. Peaks which are close (typically within about twice the resolution of the data) to an existing heavy-atom site or its symmetry equivalent are not considered. New solutions which are equivalent to any solution which has been examined previously from this seed are ignored. Each trial solution is then refined and scored.

Once a solution with a number of heavy-atom sites has been constructed, the solution as a whole may contain enough information to show that one or more of the sites included at an early stage are not correct. *SOLVE* identifies these in several ways. One is that the incorrect sites may refine to zero occupancy during heavy-atom refinement and be deleted. Another way is to systematically delete each site in a solution and test whether the solution lacking the site has a higher score than the original. *SOLVE* ordinarily carries out this deletion procedure on all trial solutions.

Finally, *SOLVE* attempts to generate additional trial solutions by inversion of all the heavy-atom sites in the seed. The reason this is useful is that three of the four scoring criteria will yield identical results for a solution and its inverse even if anomalous differences have been measured (as long as the space group is not chiral). The Patterson analysis, the cross-validation difference Fourier analysis and the figure of merit are all independent of the hand of the solution for achiral space groups. Of our four criteria, only the native Fourier analysis can distinguish the hand of the heavy atoms in this case, and then only if anomalous differences are included in the analysis. This means that in early stages of generating the heavy-atom solution, where the native Fourier is very noisy and contributes little to the scoring, it is difficult to identify the correct hand of the heavy atoms. Consequently a solution may be built up that is largely correct but has the wrong hand. Therefore, *SOLVE* tests the inverse of each heavy-atom solution in an attempt to generate a solution with the correct hand when anomalous differences are used and the space group is achiral.

### 2.18. Restricting the heavy-atom search once a promising partial solution is found

If *SOLVE* does not find any solutions which are very likely to be correct, it begins with each seed in turn and attempts to complete it as described above. On the other hand, if a very promising partial solution is found, *SOLVE* will just attempt to complete it as quickly as possible and finish. *SOLVE* uses a simple set of criteria to identify promising solutions. They must have an overall figure of merit of 0.5 or greater and an overall *Z* score of 10 or greater (that is, it must be about 10 standard deviations above the average score of starting solutions obtained from *HASSP*). When *SOLVE* finds such a solution, it no longer generates trial solutions by single-site deletions and it only keeps the one top solution present at any time (instead of a group of top solutions). Once this solution cannot be further improved by addition of new sites found in difference Fourier analyses, *SOLVE* once again tests solutions generated both by deletion and addition. When no further improvement is obtained in this way, the highest-scoring solution is reported.

### 2.19. Calculating native phases

Native phases are needed for calculation of electron-density maps as well as for three of the four criteria used in scoring (cross-validations, difference Fouriers, figure of merit and analysis of the native Fourier). In all cases, *SOLVE* uses the ‘best’ rather than ‘most probable’ phases for analysis (Blundell & Johnson, 1976 ▶). For MIR data, Bayesian correlated phasing (Terwilliger & Berendzen, 1996 ▶) is used at all stages of *SOLVE* operation. This phasing approach automatically takes into consideration any correlated non-isomorphism or errors in the derivative data. For MAD or SIRAS data, phasing during the heavy-atom solution phase of *SOLVE* operation is carried out using a standard approach as implemented in the program *HEAVY* (Terwilliger & Eisenberg, 1983 ▶). For MAD data, this phasing method is much more rapid than a more complete treatment of the phasing would be (*e.g.* Terwilliger & Berendzen, 1997 ▶; de la Fortelle & Bricogne, 1997 ▶) and is useful in speeding up the operation of *SOLVE*. Once a final solution has been obtained by *SOLVE*, however, phases are calculated for MAD data using Bayesian correlated MAD phasing (Terwilliger & Berendzen, 1997 ▶), an approach which uses all the original MAD data and includes correlations of errors among the data collected at different wavelengths.

### 2.20. Output of SOLVE

The final output of the *SOLVE* algorithm consists of an electron-density map (in *newezd* format compatible with *O*; Jones *et al.*, 1991 ▶), which can be imported into the *CCP*4 suite (Collaborative Computational Project, Number 4, 1994 ▶) using the routine *mapman*, a file containing native structure-factor amplitudes, phases and Hendrickson–Lattman coefficients (Hendrickson & Lattman, 1979 ▶), which can be imported into the *CCP*4 suite using *f2mtz*, and a command file which can be modified and used to run *SOLVE* and calculate phases or generate additional heavy-atom sites.

### 2.21. Generation of model X-ray data sets

*SOLVE* can model raw X-ray data for either MIR or MAD in which the macromolecular structure is defined by a file in PDB format (Bernstein *et al.*, 1977 ▶) and heavy-atom parameters are specified by the user. The generate feature allows any degree of ‘experimental’ uncertainty in measurement of intensities. It also allows limited non-isomorphism for MIR data in which cell dimensions differ for native and any of the derivative data sets (but in which the macromolecular structure is identical).

Once a data set has been generated, the *SOLVE* algorithm then can be applied to the data set in an attempt to solve it. *SOLVE* can calculate an electron-density map based on the structure input in PDB format and evaluate the correlation coefficient of this map with the maps that it generates during the structure-determination process. For heavy-atom solutions with the inverse hand, this comparison is of course not possible. For heavy-atom solutions which are related to a different origin than the correct solution, the origin shift is automatically determined by *SOLVE* by finding the origin shift which leads to the closest correspondence of heavy-atom sites in the trial and correct solutions. We use this correlation coefficient as an objective measure of the quality of a heavy-atom solution and as a basis for evaluating the utility of our four scoring criteria.

Model data sets were constructed using the ‘generate’ feature of *SOLVE*, using two different model proteins. One model protein consisted of coordinates from a dehalogenase enzyme from *Rhodococcus* species ATCC 55388 (American Type Culture Collection, 1992 ▶), determined recently in our laboratory, containing 316 amino-acid residues and crystallizing in space group *P*2_1_2_1_2 with cell dimensions *a* = 94, *b* = 80, *c* = 43 Å (J. Newman, personal communication). The other was based on the gene 5 protein structure in space group *C*2 with cell parameters *a* = 76, *b* = 28, *c* = 42 Å, β = 103° (PDB entry 1bgh; Skinner *et al.*, 1994 ▶). For the MIR data ‘experimental’ uncertainties of 3–5% (on intensity) and variation in cell dimensions of 1% from crystal to crystal were used. For the MAD data uncertainties of 2–4% were used. The dehalogenase model was used to generate 132 MIR data sets consisting of a native crystal and two derivative crystals. Each MIR data set contained 6–10 Hg or Au heavy-atom sites with ‘occupancies’ of 0.4–2.6 and thermal factors of 30–50 Å^2^ (although the higher values of ‘occupancy’ are not realistic for this structure, they are included to simulate the effects of a full occupancy Hg or Au in a smaller structure). The gene 5 protein model was used to generate 287 MAD data sets with 4–8 selenomethionine sites with ‘occupancies’ of 0.6–1.4 and thermal factors of 30–50 Å^2^. All the data sets were generated including anomalous differences. During the course of each structure determination, trial solutions were scored using the four criteria in Table 1 ▶. The *Z* scores for each trial solution and the correlation coefficients of trial and correct electron-density maps were recorded for all trial solutions which had the correct hand. Those that had the opposite hand were not considered, as our simple correlation-coefficient measure of the actual quality of solutions was not applicable.

## 3. Results

### 3.1. A scoring system for evaluating heavy-atom partial structures in the MAD and MIR methods

The approach we have taken for evaluating MIR heavy-atom (or anomalously scattering atom in the MAD method) partial structures is to quantify criteria that have been applied in a qualitative fashion for some time in the MIR and MAD approaches. The first criteria (Table 1 ▶) is the match between the Patterson function and the interatomic vectors predicted from the trial heavy-atom structure (Blundell & Johnson, 1976 ▶). The second consists of the peak heights at heavy-atom positions in ‘cross-validation’ difference Fourier maps. These are calculated by using all but one heavy atom in phasing. The peak height at the position of the deleted atom is a measure of the self-consistency of the heavy-atom solution (Dickerson *et al.*, 1961 ▶). The third criteria we use is simply the figure of merit of phasing (Blundell & Johnson, 1976 ▶). This is a measure of the precision of the phases obtained. The final criteria is the existence of well defined regions containing solvent and macromolecule in the native electron-density map (Terwilliger & Berendzen, 1999 ▶). These criteria are described in detail in §[Sec sec2]2.

### 3.2. Evaluating scoring criteria using *SOLVE* to generate and analyze model data

To evaluate the scoring criteria illustrated in Table 1 ▶ and to test the overall *SOLVE* algorithm, model data were constructed using the ‘*generate*’ feature of *SOLVE* based on crystal structures of a dehalogenase enzyme (J. Newman, personal communication) and gene 5 protein (Skinner *et al.*, 1994 ▶). The *SOLVE* structure-solution algorithm was then applied to these model data sets and the utility of the scoring criteria was evaluated by comparing them with the correlation coefficient between maps calculated by *SOLVE* during structure determination and model maps.

### 3.3. Evaluating *SOLVE* scoring criteria

Each of our four scoring criteria was evaluated for a series of 419 model structure determinations using the correlation coefficient between correct and trial electron-density maps as a measure of the actual quality of each solution. The purpose of this comparison is to evaluate whether the four scoring criteria are useful in differentiating between solutions which lead to a map of high quality and those which do not.

**Figure 3:**
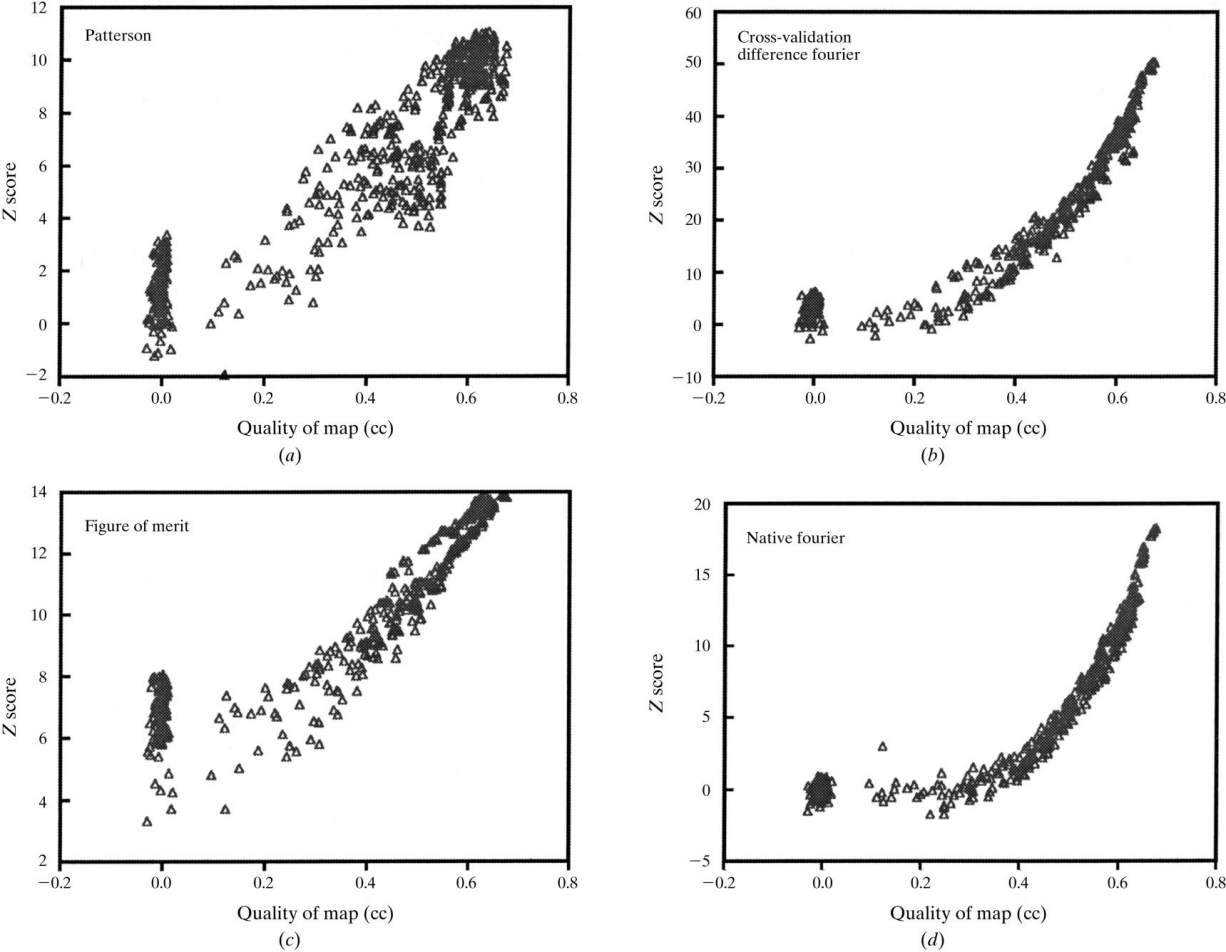
*Z* scores for one model structure determination. Each point correponds to one trial heavy-atom partial structure. The *x* axis is the quality of the solution (the correlation coefficient of the map calculated using the trial heavy-atom structure with the true map). The *y* axis is the *Z* score for the scoring criterion. The scoring criteria shown are (*a*) agreement with the Patterson function, (*b*) the cross-validation difference Fourier analysis, (*c*) the figure of merit of phasing and (*d*) the distinction between solvent and protein regions in the native electron-density map.

Fig. 3 ▶ shows the *Z* scores for each scoring criterion for one of the 419 test cases (based on the dehalogenase and gene 5 protein structures) as a function of the quality of the solutions (the correlation coefficient of the corresponding electron-density map to the model map). As expected, the *Z* scores for each criterion generally increase with increasing correlation coefficients between model and trial maps. The relationship between correlation coefficient and *Z* scores differs considerably from one criterion to another, however. The *Z* scores for agreement with the Patterson function increase gradually over the range of correlation coefficients. In contrast, the *Z* scores for cross-validation Fourier analyses are nearly constant over the range of correlation coefficients from 0 to 0.25, but then increase at a much greater rate than the Patterson scores.

Fig. 3 ▶ indicates that any of the four criteria we have selected would have some use in evaluating the relative quality of different trial solutions, but that the different criteria have slightly different behavior at different stages of structure determination. In particular, the Patterson analysis and cross-validation Fourier analyses appear to be of the most use for solutions with correlation coefficients in the range 0.3–0.4, while the analysis of the native Fourier appears to be the strongest criterion for identification of correct solutions with correlation coefficients above this range.

**Figure 4:**
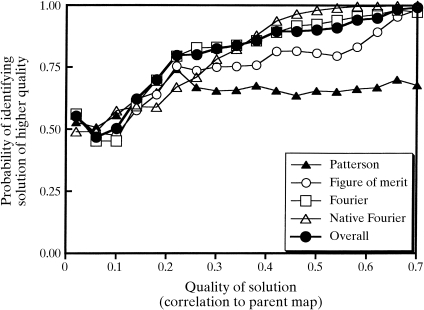
Probability of identifying the better of two trial heavy-atom solutions. Automated structure solutions were carried out on 419 model data sets. For each trial heavy-atom solution, the individual *Z* scores for each scoring criteria and the overall overall *Z* score were noted. The quality of the map, based on the correlation coefficient of the native electron-density map to the model map, was recorded as well. All pairs of heavy-atom solutions for a single model data set which differed in quality (correlation coefficient) by 0.05 ± 0.02 were then examined to determine whether the solution with the higher *Z* score had the higher correlation coefficient. The percentage of cases in which the *Z* score correctly identified the solution with the higher correlation coefficient is plotted as a function of the correlation coefficient of map to model map obtained from the solutions.

One way to illustrate the predictive power of each criterion is to evaluate its ability to determine which of two possible solutions that differ in quality by a certain amount (*e.g.* 0.05 units of correlation coefficient between model and trial maps) is of a higher quality. This ability is central to the *SOLVE* algorithm, which maintains a ranked list of top solutions at any one time. This probability can be estimated from Fig. 3 ▶ by determining the percentage of cases where the solution with the higher correlation coefficient has a higher score. Pairwise comparisons of solutions which differed by 0.05 units in correlation coefficient were used in this analysis. Fig. 4 ▶ shows a plot based on all 419 test structure determinations which illustrates this probability where all the pairwise comparisons are within the same structure determination. For solutions of poor quality (with correlations between model and trial maps of less than about 0.1) all of the criteria had only about a 50% chance of identifying the better solution in a pairwise comparison. In contrast, for solutions with better quality (with correlations between model and trial maps of about 0.3–0.5), each scoring criteria had considerable utility in identifying the better solution. Comparison of a solution with the Patterson function allowed a correct identification in about 60% of the cases. The figure of merit could be used to make this distinction in about 75% of the cases. The cross-validation difference Fourier was correct in about 80–85% of cases, and analysis of the native Fourier map each could be used in 75–95% of cases to identify the better solution. The overall *Z* score was nearly as good as the best of the four individual criteria over the entire range of map quality. Therefore, it appears to be a reasonable overall measure of the quality of a solution.

**Figure 5:**
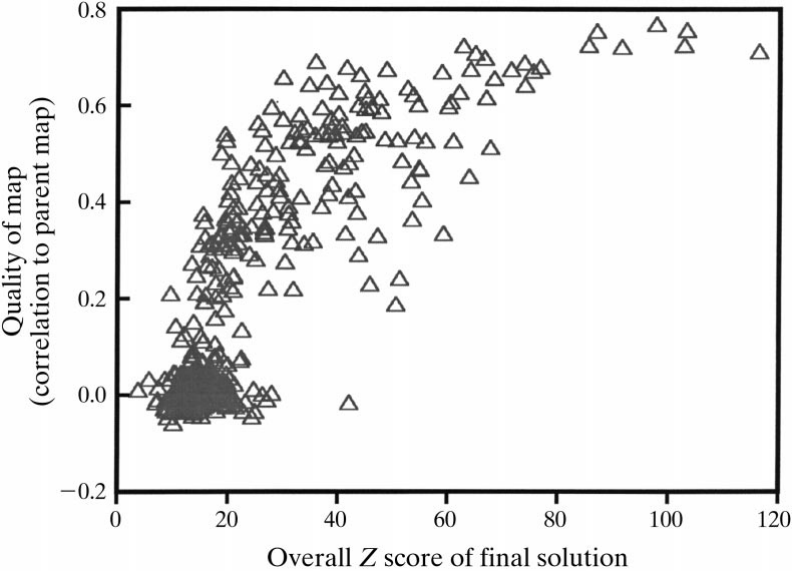
Highest *Z* scores and quality of electron-density maps for 419 model structure determinations. Each point correponds to the highest scoring solution from one model structure determination. The *x* axis is the correlation coefficient of the map calculated by *SOLVE* with the model (true) map. The *y* axis is the overall *Z* score for this solution.

After the *SOLVE* algorithm is applied to a crystal structure, it is useful to have an idea of whether the top solution that it has found is likely to actually represent a correct solution. Fig. 5 ▶ shows the overall score and correlation coefficient to the model map of the top solutions found in each of the 419 model structure determinations we carried out. In 180 of the 419 structure determinations shown in Fig. 5 ▶, *SOLVE* was able to obtain an electron-density map with a correlation coefficient to the model map of 0.2 or greater. Fig. 5 ▶ indicates that in this set of test-structure determinations with 4–10 heavy-atom sites those solutions with overall *Z* scores of greater than 20 were nearly always correct. Those with scores in the range of about 10–20 were sometimes correct and sometimes not, and those with scores less than 10 were rarely correct. It should be noted that although these results with model data give a general idea of the range of scores which are associated with maps of various qualities, the relationship between map quality and overall scores is likely to be dependent on the details of the structure determination. Consequently, Fig. 5 ▶ should be used only as a rough guide to the likely quality of a solution.

### 3.4. Application of *SOLVE* to experimental MAD and MIR data

*SOLVE* has now been used to determine many MIR and MAD structures, with two of the largest structures consisting of MAD structures with 26 and 52 selenomethionine residues in the asymmetric unit, respectively (S. Ealick, personal communication; W. Smith & C. Janson, personal communication). A test MAD structure determination (with 15 selenomethione sites in the asymmetric unit) and an actual MIR structure deterimination (with five derivatives, each containing 2–4 heavy-atom sites) are illustrated here to evaluate the application of *SOLVE* to experimental data.

### 3.5. MAD structure determination

A four-wavelength MAD data set collected on β-catenin (Huber *et al.*, 1997 ▶) was used to test *SOLVE* on MAD data. This structure was originally solved using *RSPS* (Knight, 1989 ▶), but it was a good test case because of the large number of selenomethione residues (15) in the protein and the availability of a refined structure for comparison. The space group was *C*222_1_ with unit-cell dimensions of *a* = 64, *b* = 102, *c* = 187 Å. Scaled MAD data (17000 observations to a resolution of 2.7 Å) was converted to intensity data. This reflection information was input to *SOLVE* along with the approximate number of amino-acid residues in the protein (700), the number of expected selenium sites (15) and estimates of the scattering factors for selenium (*SOLVE* can refine the values of the scattering factors if they are not known accurately). Default values were used for all other parameters. *SOLVE* identified a single solution with 12 selenium locations. All 12 selenium locations as well as the hand of the solution were correct. The additional selenium sites used in the original structure determination included one with a thermal factor of 85 Å^2^ and two with partial occupancies in the refined structure (Huber *et al.*, 1997 ▶). The overall figure of merit of the MAD phasing was 0.67 and the overall *Z* score of the solution was 54. *SOLVE* required approximately 4 h on a 500 MHz DEC Alpha workstation to find this solution, and three additional hours to verify that no similar solutions would yield higher overall scores.

**Figure 6:**
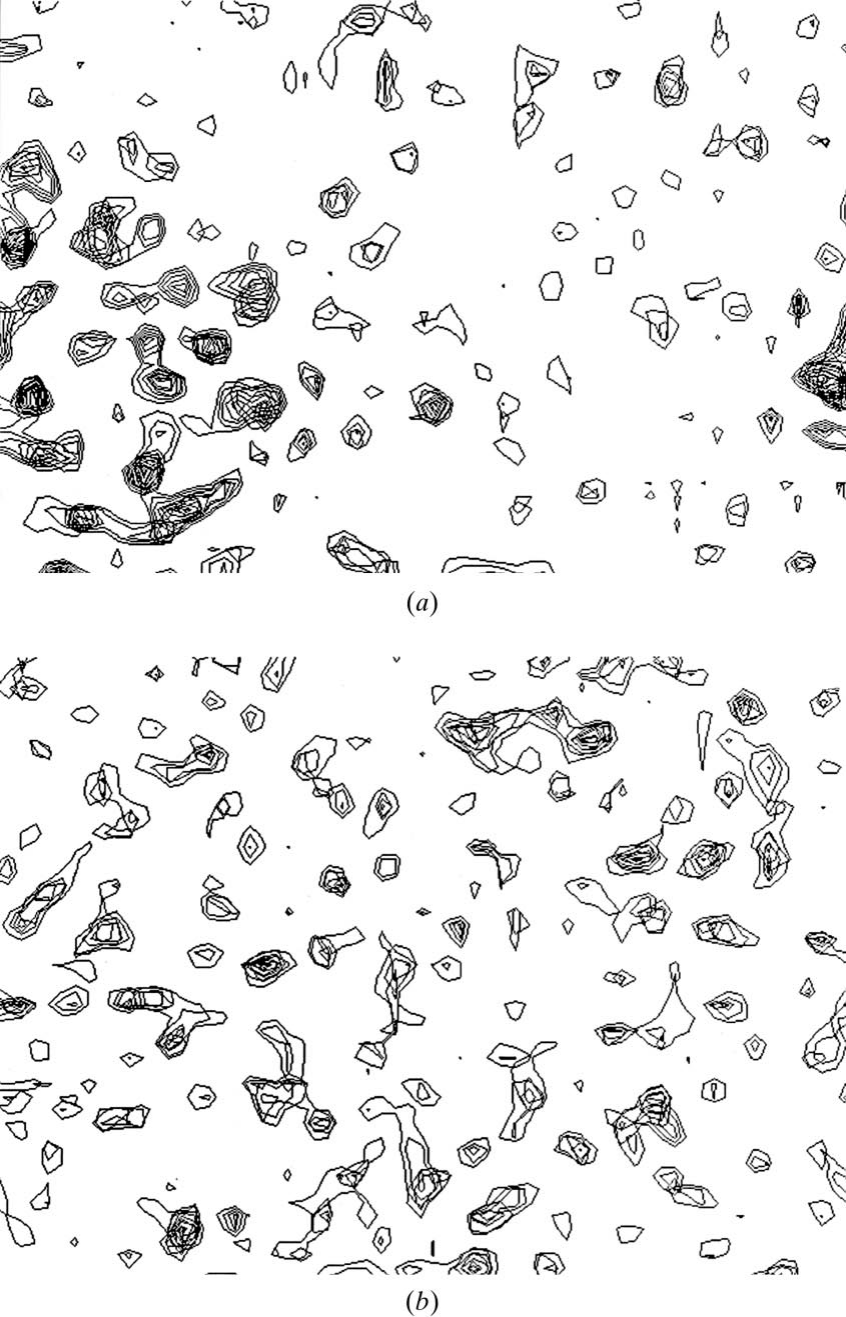
Identification of hand of selenium partial structure using the native Fourier. (*a*) Section through an electron-density map of β-catenin calculated using 11 correct selenium sites. (*b*) As (*a*), but with inverted hand of Se atoms.

The hand of the selenium partial structure was identified by *SOLVE* using the analysis of the native Fourier map. The *Z* score for analysis of the native Fourier for the correct hand was 4.7 (*i.e.* the final solution had a score 4.7 standard deviations above the starting set of trial solutions), while that of the inverse hand was only 0.5. The utility of this analysis of the native Fourier map is illustrated in Fig. 6 ▶, which shows sections through the native Fourier calculated by *SOLVE* using 11 selenium sites with either the correct or inverted hands. The map with the correct hand has features expected of a protein: regions which are flat (solvent) and other regions which have high variation (the protein). In contrast, the map calculated with an inverted set of selenium sites has a very uniform level of variation throughout and does not have the appearance expected of a protein crystal.

**Figure 7:**
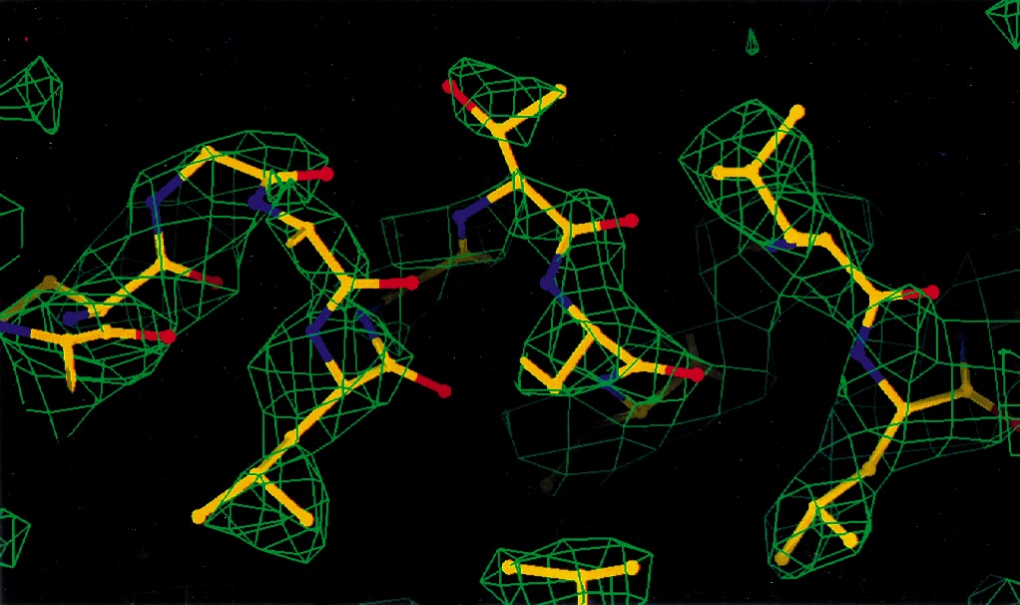
*SOLVE* electron-density map of β-catenin.

Fig. 7 ▶ shows a section of electron density from the map calculated by *SOLVE* and coordinates from the refined model of β-catenin (with an origin shift so that the selenium sites used in the original structure determination match the ones obtained by *SOLVE*). The electron-density map is of high quality and is readily interpretable.

### 3.6. MIR structure determination with *SOLVE*

**Figure 8:**
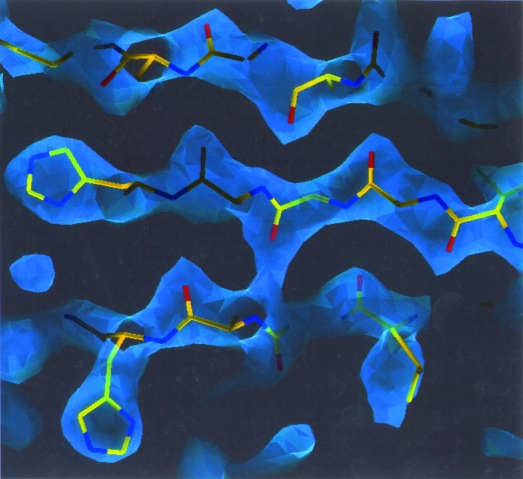
*SOLVE* electron-density map of *Rhodococcus* dehalogenase.

*SOLVE* was recently used in the structure determination of a dehalogenase enzyme from *Rhodococcus* strain ATCC 55388 (J. Newman, personal communication). This protein crystallized in space group *P*2_1_2_1_2 with cell parameters of *a* = 94, *b* = 80, *c* = 43 Å, and MIR data was collected to a resolution of 2.5 Å on the native and five derivatives. Raw unmerged data produced by *HKL* (Otwinowski & Minor, 1997 ▶) was input to *SOLVE*, along with the identities of the heavy atoms in each derivative, a limit of five heavy-atom sites per derivative and the estimated number of amino-acid residues in the asymmetric unit (250). *SOLVE* identified between two and four heavy-atom sites in each derivative and calculated the electron-density map illustrated in Fig. 8 ▶, which has an overall figure of merit of 0.69. The map is of excellent quality and is readily interpretable. For the actual structure solution, this map was further improved by solvent flattening (Abrahams *et al.*, 1994 ▶). This structure determination required approximately 4 h to obtain and 1 h to check using a 500 MHz DEC Alpha workstation.

## 4. Conclusions

We have found the *SOLVE* algorithm to be exceptionally useful in determining macromolecular structures based on MIR and MAD X-ray data, both because of its simplicity of use and in the thoroughness of its search for heavy-atom solutions. The simplicity of using *SOLVE* is largely made possible by the development of quantitative measures of the quality of heavy-atom solutions, allowing the determination of the heavy-atom structure to be transformed from a decision-making problem with incompletely defined criteria to a straightforward optimization problem. Simplicity of use is also made possible by choosing default parameters which are applicable to a wide variety of situations, so that in most cases it is not necessary for the user to adjust them. The incorporation of robust yet standardized methods for scaling is also important for the ease of use of *SOLVE*, as it is therefore able to begin with raw data files containing integrated intensities and scale MIR or MAD data without manual intervention.

The thoroughness of the search for heavy-atom solutions is an important feature of *SOLVE*. In the MIR method, a search for a ‘good’ (usually single-site) derivative with which to find the heavy-atom sites in all the other derivatives is often a time-consuming and difficult stage in structure determination. In this process, tools such as *RSPS* (Knight, 1989 ▶) or *HASSP* (Terwilliger *et al.*, 1987 ▶) are often used to generate plausible solutions to a difference Patterson function. These solutions must then be individually checked for their agreement with the Patterson and their ability to contribute to phasing the native data and to identify heavy-atom sites in other derivatives. As the process is often slow and involved, only a small number of solutions usually can be tested. Because *SOLVE* is automated, it is now practical to test many more starting solutions and to follow each one through, building up complete trial MIR solutions which can be evaluated relative to each other using the objective *SOLVE* scoring system. Using this scoring system, the correctness of each individual heavy atom in the solution can also be checked by deleting it and re-evaluating the score of the solution.

One of the most important features of *SOLVE* is its ability to evaluate the quality of an electron-density map during the structure-determination process and to use this as part of the evaluation of each trial heavy-atom solution. When MIR or MAD heavy-atom structures are determined using either the Patterson function (Terwilliger *et al.*, 1987 ▶; Chang & Lewis, 1994 ▶) or by direct methods (Sheldrick, 1990 ▶; Miller *et al.*, 1994 ▶), structure-factor amplitudes corresponding to the heavy-atom partial structure are extracted from the raw data. Because of this separation of heavy-atom structure factors from total structure factors, information contained in the original structure factors which could be used to solve the heavy-atom partial structure is ignored. In particular, only after the heavy-atom partial structure is ‘solved’ is a native Fourier calculated and visually examined. In contrast, *SOLVE* is able to evaluate potential heavy-atom solutions both with respect to their agreement with the Patterson function and with respect to the qualities of the resulting native Fourier, cross-validation difference Fourier and figure of merit. The examination of the native Fourier not only yields information on the overall quality of a solution but also can often positively identify the hand of the heavy-atom solution when anomalous differences have been measured. The incorporation of these different sources of information about the quality of heavy atom solutions allows *SOLVE* to use more of the information present in a MAD or MIR experiment than has previously been possible during the process of structure determination.

*SOLVE* is fundamentally different from other software used for MIR and MAD structure determinations because of its incorporation of quantitative measures of the quality of a solution and because of its complete automation. Other packages such as *PHASES* (Furey & Swaminathan, 1997 ▶), *HEAVY* (Terwilliger & Eisenberg, 1987 ▶; Terwilliger & Berendzen, 1996 ▶) or *SHARP* (de la Fortelle & Bricogne, 1997 ▶) can carry out all the steps necessary to determine a structure by MIR or MAD, but they do not provide the range of objective and quantifiable measures of the quality of a potential solution that *SOLVE* does. A user must for example evaluate a native Fourier map visually to assess whether a solution is likely to be correct. Because of its ability to provide quantitative measures of the quality of a solution, *SOLVE* is both able to provide the user with useful criteria for comparing solutions when the user wishes to be closely involved in decision making in the structure-determination process, and it is able to carry out the entire process without any input at all.

We anticipate that *SOLVE* will be of significant use not just in MAD and MIR structure determinations carried out one-by-one as they are today, but also in more high-throughput applications which are now being widely discussed. Because of the automation and ease of use of *SOLVE*, it has already been used in several instances to solve a structure within a few hours of the data being collected (R. Fahrner & D. Eisenberg, personal communication; R. Stevens, personal communication). It seems reasonable to imagine a largely automated process of structure determination at synchrotron sources beginning with MAD data collection (*e.g.* on selenomethionine-containing crystals) and continuing through data processing and structure solution at least as far as calculation of an electron-density map. With further development of automated model building and refinement (Zou & Jones, 1996 ▶), the entire process of structure determination and model building and refinement might be automated for straightforward cases. For more complicated cases which cannot be solved automatically, the quantitative evaluation of heavy-atom solutions carried out by *SOLVE* is likely to be an important tool for the macromolecular crystallographer in structure determination.

Complete documentation of the *SOLVE* software and information on obtaining the program are available on the internet at http://www.solve.lanl.gov.

## Figures and Tables

**Table 1:** Criteria for evaluation of MIR and MAD heavy-atom partial structures

Agreement with Patterson function
Cross-validation difference Fourier
Figure of merit
Defined solvent and macromolecule in native Fourier
